# A targeted next‐generation sequencing in the molecular risk stratification of adult acute myeloid leukemia: implications for clinical practice

**DOI:** 10.1002/cam4.969

**Published:** 2017-01-10

**Authors:** Po‐Han Lin, Huei‐Ying Li, Sheng‐Chih Fan, Tzu‐Hang Yuan, Ming Chen, Yu‐Hua Hsu, Yu‐Hsuan Yang, Long‐Yuan Li, Su‐Peng Yeh, Li‐Yuan Bai, Yu‐Min Liao, Chen‐Yuan Lin, Ching‐Yun Hsieh, Ching‐Chan Lin, Che‐Hung Lin, Ming‐Yu Lien, Tzu‐Ting Chen, Yen‐Hsuan Ni, Chang‐Fang Chiu

**Affiliations:** ^1^ Department of Medical GeneticsNational Taiwan University HospitalTaipeiTaiwan; ^2^ Graduate Institute of Clinical MedicineChina Medical UniversityTaichungTaiwan; ^3^ Department of Genomic MedicineCenter for Medical GeneticsChanghua Christian HospitalChanghuaTaiwan; ^4^ Department of Life SciencesNational Chung Hsing UniversityTaichung40227Taiwan; ^5^ Division of Hematology and OncologyDepartment of Internal MedicineChina Medical University Hospital; ^6^ Department of Internal MedicineGraduate Institute of Clinical MedicineChina Medical UniversityTaichungTaiwan; ^7^ Department of PediatricsNational Taiwan University HospitalTaipeiTaiwan

**Keywords:** Acute myeloid leukemia, gene mutations, next‐generation sequencing, precision medicine, prognosis

## Abstract

Conventional cytogenetics can categorize patients with acute myeloid leukemia (AML) into favorable, intermediate, and unfavorable‐risk groups; however, patients with intermediate‐risk cytogenetics represent the major population with variable outcomes. Because molecular profiling can assist with AML prognosis and next‐generation sequencing allows simultaneous sequencing of many target genes, we analyzed 260 genes in 112 patients with *de novo* AML who received standard treatment. Multivariate analysis showed that karyotypes and mutation status of *TET2*,*PHF6*,*KIT*, and *NPM1* ^mutation^/*FLT3*‐ internal tandem duplication (ITD)^negative^ were independent prognostic factors for the entire cohort. Among patients with intermediate‐risk cytogenetics, patients with mutations in *CEBPA* ^double mutation^, *IDH2*, and *NPM1* in the absence of *FLT3*‐ITD were associated with improved Overall survival (OS), similar to those with favorable‐risk cytogenetics; patients with mutations in *TET2*,*RUNX1*,*ASXL1*, and *DNMT3A* were associated with reduced OS, similar to those with unfavorable‐risk cytogenetics. We concluded that integration of cytogenetic and molecular profiling improves prognostic stratification of patients into three groups with more distinct prognoses (*P *<* *0.001) and significantly reduces the number of patients classified as intermediate risk. In addition, our study demonstrates that next‐generation sequencing (NGS)‐based multi‐gene sequencing is clinically applicable in establishing an accurate risk stratification system for guiding therapeutic decisions.

## Introduction

Acute myeloid leukemia (AML) is a heterogeneous disease that is characterized by impaired differentiation and increased proliferation of immature myeloid cells. For adult AML patients receiving intensive treatment, the 5‐year survival rate is only ~30% 1. The prognosis of AML patients can be classified as favorable, intermediate, and unfavorable groups according to recurrent cytogenetic and genetic abnormalities 2, 3. Patients with a cytogenetic profile that includes the translocation of *PML*‐*RARA* [*t*(15;17) (q24;q21)], *RUNX1*‐*RUNX1T* [*t*(*8*;*21*)(q22;q22)], or *CBFB*‐*MYH11* [inv(16) or t(16;16)(p13.1;q22)] are classified as favorable‐risk group and have good outcomes with chemotherapy‐based consolidation treatment 4, 5. Patients with complex cytogenetic changes are classified as unfavorable group and have a poor prognosis 5. Allogeneic hematopoietic stem cell transplantation (HSCT) may be required to improve the outcome of the unfavorable‐risk patients 6. However, half of AML patients belong to an intermediate‐risk group, and most of their leukemia has normal karyotypes 5. Recent translational researches show that mutation profiling of several genes, including *FLT3*,* NPM1*,* KIT*,* RAS*,* CEBPA*,* IDH1*,* IDH2*, and *TET2*, provides prognostic prediction and treatment guidance for patients with normal karyotypes 2, 3, 7, 8, 9. For example, patients with *NPM1* mutation without *FLT3*‐internal tandem duplication (ITD) have a favorable prognosis 10, whereas patients with *AXSL1* or *TET2* mutation have a poor prognosis 11, 12. For accurate risk stratification, the current consensus suggests that cytogenetic and genomic mutation analyses should be integrated for prognostic and therapeutic decisions regarding AML patients 3.

Because next‐generation sequencing (NGS) technology enables parallel analysis of many genes, NGS is used not only in research but also in clinical molecular diagnosis 13, 14. This strategy may solve the challenges of multiple gene screening from conventional platforms. However, the number of genes that should be screened for AML patients is not clear, and it would be beneficial to know whether NGS could define a new genetic mutation profile to serve as a prognostic indicator in AML patients. Previous whole‐genome and exome analyses have demonstrated recurrent mutations in 260 genes in 200 AML patients 15; however, the prognostic impact of these genes remains unclear. Therefore, we used a sequencing panel containing these 260 genes to screen mutations in the 112 patients. First, we demonstrated the clinical feasibility of NGS to the molecular diagnosis of AML. Second, we searched for novel prognostic factors and would like to establish a precise molecular classification based on the integration of cytogenetic and molecular alterations.

## Materials and Methods

### Patients

The diagnosis of AML was based on the definition of World Health Organization. All of the enrolled patients received standard chemotherapy with or without allogeneic HSCT as previously described. The diagnosis of AML was based on the World Health Organization definition, and all of the enrolled patients received standard chemotherapy with or without allogeneic HSCT as described 16. The mononuclear cells of each bone marrow sample were also collected and cryopreserved in the biobank after the patients had signed informed consent. This study was approved by the Institutional Review Board of China Medical University Hospital (DMR101‐IRB2‐020).

### Constructing a shotgun genomic sequencing library

Genomic DNA (gDNA) was isolated from bone marrow mononuclear cells using the QIAGEN Genomic DNA extraction kit. The purities and concentrations of gDNA were confirmed by electrophoresis, Nanodrop 2000 (Thermo Scientific, USA), and a Qubit 2.0 Fluorometer (Life Technologies, USA). Double‐stranded DNA (dsDNA; 2 *μ*g) that passed the quality‐control steps was sheared to ~300 bp with an M220 focused ultrasonicator (Covaris, USA). Size distribution of the fragmented DNA was confirmed using a Bioanalyzer 2100 (Agilent Technologies, USA), and shotgun genomic libraries for use with the MiSeq platform (Illumina, USA) were generated using the KAPA Library Preparation kit (Kapa Biosystems, USA) according to the manufacturer's protocol.

### Capture‐based NGS

To test if capture‐based target‐enrichment NGS is applicable, AML genetic testing, the xGen^®^ AML Cancer Panel v1.0 containing 11,743 xGen Lockdown^®^ probes was purchased from Integrated DNA Technologies (USA) which targeted important exons of the AML disease pathway related genes (Table S1) 15. A total of ~1.2 Mbp of gDNA target regions from 6235 exons of genes related to AML were used to design probes. For each capture reaction, multiplex libraries containing 13 libraries pooled equally were used for probe hybridization, and target enrichment was performed according to the Integrated DNA Technologies—optimized xGen 4‐h capture protocol. The libraries were then purified with AMPure XP beads for MiSeq sequencing.

### MiSeq high‐throughput sequencing and data processing

The concentrations of captured libraries were determined by real‐time quantitative PCR with Illumina adapter‐specific primers provided with the KAPA Biosystems library quantification kit. Libraries were denatured and sequenced on the MiSeq platform with v2 reagent for paired‐end sequencing (2 × 150 bp). Instrument control, cluster generation, image capture, and base calling were processed with Real Time Analysis software 1.18.54, MiSeq Control software 2.4.1.3, and MiSeq Report software 2.4.60.8. FASTQ files generated by MiSeq Report were used for further analysis.

The post‐NGS bioinformatics was described previously 17. The FASTQ files were aligned to the human reference genome (February 2009, GRCh37/hg19) using the BWA‐MEM algorithm in BWA software (version 0.7.4) 18. Picard tools (version 1.90) were used to perform the necessary data conversion, sorting, and indexing 19. GATK software (version 2.5‐2) was used for variant identification including the UnifiedGenotyper and HaplotypeCaller tools for variant calling and the VariantFiltration tool for variant filtration 20. Gene annotation, amino acid change annotation, SIFT and PolyPhen2 scores, dbSNP identifiers (dbSNP 138), 1000 Genomes Project allele frequencies, and NHLBI‐ESP 6500 exome project allele frequencies of filtered variants were annotated with ANNOVAR (2014‐OCt) 21. In addition to the analysis mentioned above, BAM files were further analyzed by Pindel (version 0.2.4) for *FLT3*‐ITD identification (Figure S1) 22, 23, 24, 25.

### Variant filtration

After annotations, variants were interpreted mainly based on ACMG guideline 26. The frameshift insertion or deletion (indel) variants, nonsense variants, and splice‐site variants with allele frequencies <1% in both the 1000 Genomes Project and NHLBI‐ESP 6500 exome project were included for further analysis. SIFT and PolyPhen2 scores were used to evaluate the effects of specific missense variants on the protein 27, 28; only missense variants with scores >0.95 in PolyPhen2 and SIFT scores <0.05 were included for further analysis. However, due to lack of germline data, the rare germline variants may be falsely considered as the tumor mutations. Therefore, we used Taiwan genomics data (*N* = 997, https://taiwanview.twbiobank.org.tw/search) to exclude the germline variants which are presented in Taiwan population but rare in Western people.

In addition, previous studies had well established, the definition of pathogenic mutations of *NPM1*,* FLT3*, and *CEBPA* 29, 30, 31. The four nucleotide insertion in exon12 of NPM1 results in dis‐localization of NPM1^30^. Both internal tandem duplication and D835 mutation in FLT3 cause activated transduction signaling 29. The genetic variant of CEBPA is usually a nonframeshift insertion or deletion and the pathogenic mutations are commonly located at transactivation domain (TAD) 1, 2 and basic region mediating DNA binding leucine zipper (bZIP) region. A common benign polymorphism is an in‐frame 6‐bp insertion (ACCCGC) in the transactivation domain 2 (TAD2), resulting in a His‐Pro duplication (HP196–197 insertion)^31^.

### Statistics

Overall survival (OS) was estimated by Kaplan–Meier analysis. The chi‐squared test and Fisher's exact test were used to calculate the significance of variances between each group. Cox proportional hazard regression analysis was used to estimate the hazard ratio (HR) of OS and corresponding 95% confidence interval (CI) for various genetic alterations. All *P*‐values are two‐sided, and *P <* 0.05 was considered as significant.

## Results

### Patient characteristics

The study's 112 patients comprised 45 females and 67 males of median age 42.6 years (range, 11.7–79.0 years). There were 5, 21, 37, 9, 21, 6, 2, 1, and 10 patients diagnosed as AML with M0, M1, M2, M3, M4, M5, M6, M7, and undetermined types, respectively, according to French–American–British classification. Based on cytogenetic data, 22 patients (19.6%) were in the favorable‐risk group, 69 (61.6%) in the intermediate‐risk group, and 21 (18.8%) in the unfavorable‐risk group at initial diagnosis. Nineteen patients received allogeneic HSCT. Table S2 lists the clinical information of the enrolled patients.

### Capture enrichment and NGS performance

For all NGS data, an average of 2.65 ± 0.33 million reads that mapped to the reference genome (*hg19*, GRCh37) were generated per patient, with ~80.1% of reads (range, 74.6–83.3%) mapping to the ~1.2‐Mbp target region. The average mean coverage for the targeted exons was 185.4 ± 23.7 (range, 108.8–263.9), and >0.2 × mean coverage was observed for >96.6% of targets; 94.9 ± 5.9% of the exons had a coverage of ≥50 reads, and the median fragment length was 194 bp (range, 165–216).

### Mutation profiling and gene–gene association

**Figure 1:**
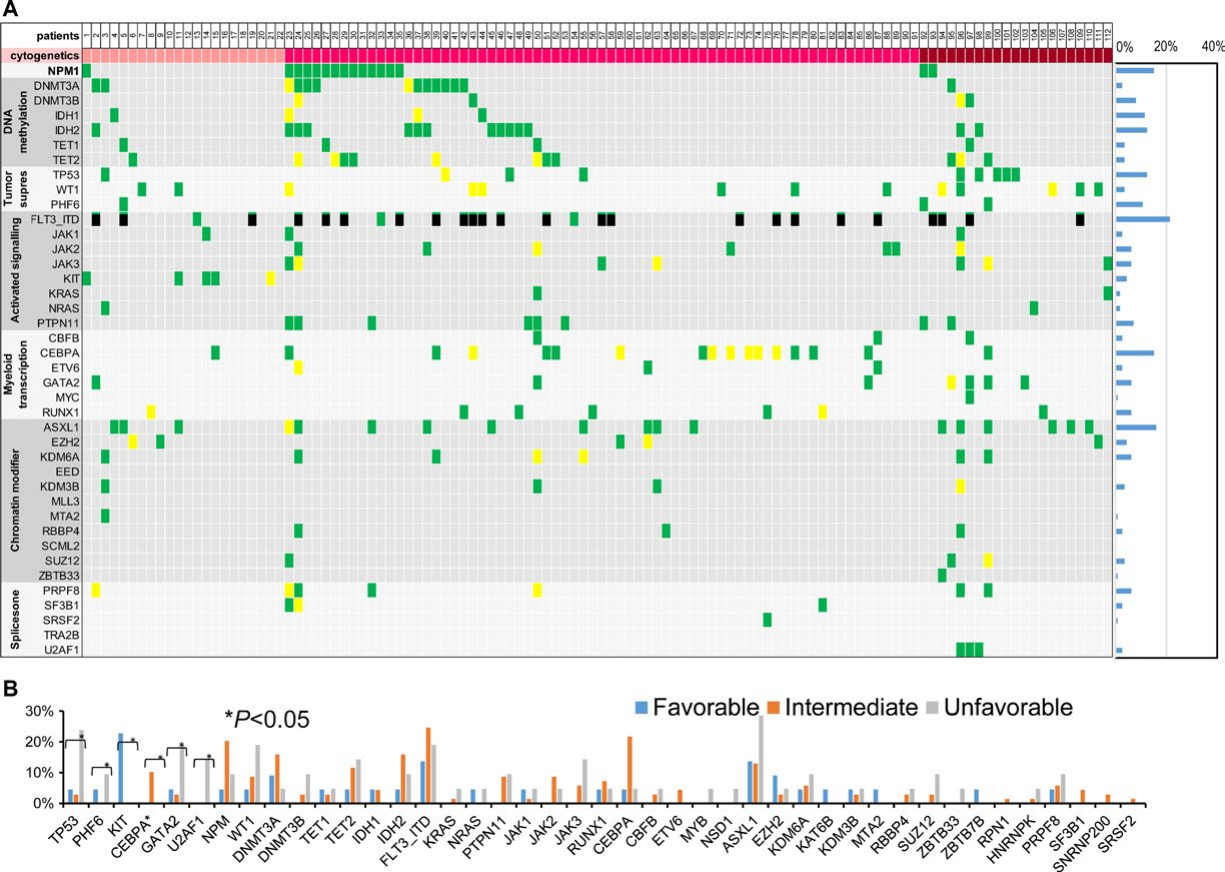
Landscape of mutations in 112 AML patients. (A) A computation plot shows cytogenetic risks and pathogenic mutations in 40 individual genes and sets of genes, grouped into seven categories, as labeled on the left. Mutation frequency for the 112 patients is illustrated in the right bar graph. For cytogenetics, pink boxes indicate favorable‐risk, red boxes indicate intermediate‐risk, and maroon boxes indicate unfavorable‐risk cytogenetics. Single mutations in a gene are labeled as green boxes, and plural mutations in a gene are labeled as yellow boxes. Each column represents data for 1 of the 112 subjects. (B) Relationship between the three cytogenetic groups and the mutation frequency of each gene. CEBPA* represents double mutation of *CEBPA* (*CEBPA* ^double mutation^), and *CEBPA* stands for incidence of all kinds of mutations. AML, acute myeloid leukemia.

Among the 260 gene analyses of the 112 patients, we identified 1926 deleterious mutations, including single‐nucleotide variants (SNVs) and small indels in coding regions, averaging 17.2 mutations per patient (range, 3–185). Forty of the 260 genes can be categorized as DNA methylation, tumor suppressor, activated signaling, myeloid transcriptional factor, chromatin modifier, and spliceosome; these genes and *NPM1* are involved in the leukemogenesis and previous studies showed that most of them might be associated with the prognosis of AML patients (Fig. 1)^3^, ^15^. Among them, the most commonly mutated gene was *FLT3*‐ITD (21.4%), followed by *ASXL1* (16.1%), *NPM1* (15.1%), *CEBPA* (15.1%), *DNMT3A* (12.5%), *IDH2* (12.5%), *WT1* (11.6%), and *TET2* (10.7%). The frequency of double allele of *CEBPA* (*CEBPA* ^double mutation^) was 6.25%. Other genes had a mutation prevalence of <10%.

Stratified by cytogenetic classification, it was a higher trend to incur mutations of the 260 genes in patients with unfavorable‐risk cytogenetics than in those with favorable‐and intermediate‐risk cytogenetics. The mutation rate of each of *TP53*,* GATA2*, and *U2AF1* was significantly higher in patients with unfavorable cytogenetics; *KIT* mutation predominated in patients with favorable‐risk cytogenetics; mutation of *CEBPA* ^double mutation^ was found in patients with intermediate‐risk cytogenetics. Among the 69 patients with intermediate‐risk cytogenetics, the most frequent mutation was *FLT3*‐ITD (*n* = 17, 24.6%), followed by mutations in *NPM1* (*n* = 13, 18.8%), *DNMT3A* (15.9%), and *IDH2* (15.9%). The frequency of *CEBPA* ^double mutation^ was 10.1%.

Pairwise mutation analysis was performed in these 40 genes to identify co‐occurring mutations and mutations that occurred exclusively (Table S3). *NPM1* mutation was found to significantly co‐occur with *FLT3, PTPN11, PRF8*, and *SF3B1* mutation. *IDH2* mutation significantly co‐occurred with mutation of *DNMT3A*,* JAK1*,* JAK2*,* JAK3*,* AXSL1*, and *U2AF1*;* IDH2* mutated exclusively with *TET2* mutation.

### Cytogenetic and genetic alterations affecting complete remission

**Figure 2:**
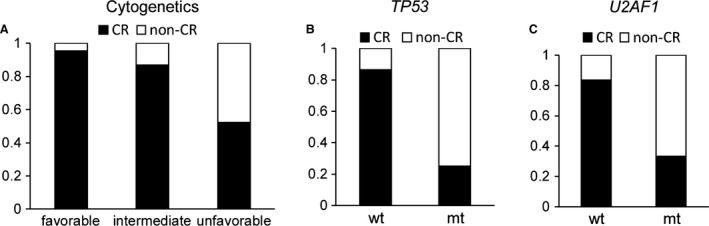
Cytogenetic and genomic lesion associations in response to induction chemotherapy. Complete remission (CR) rate stratified by (A) the three cytogenetic groups (*P *<* *0.001) and (B, C) mutation status of *TP53* (*P *<* *0.001) and *U2AF1* (*P *=* *0.025). (wt, wild type; mt, mutant.)

We assessed the value of cytogenetic and genetic mutations for predicting the remission rate of AML. Patients with favorable‐risk cytogenetics had higher complete remission (CR) rates than those with intermediate‐ and unfavorable‐risk cytogenetics (Fig. 2A, *P* < 0.001). *TP53* (*P *<* *0.001) and *U2AF1* (*P *=* *0.025) mutations were identified as unfavorable factors associated with low CR rate (Fig. 2B and C).

### Survival analysis according to cytogenetic risk and current known genes

**Figure 3:**
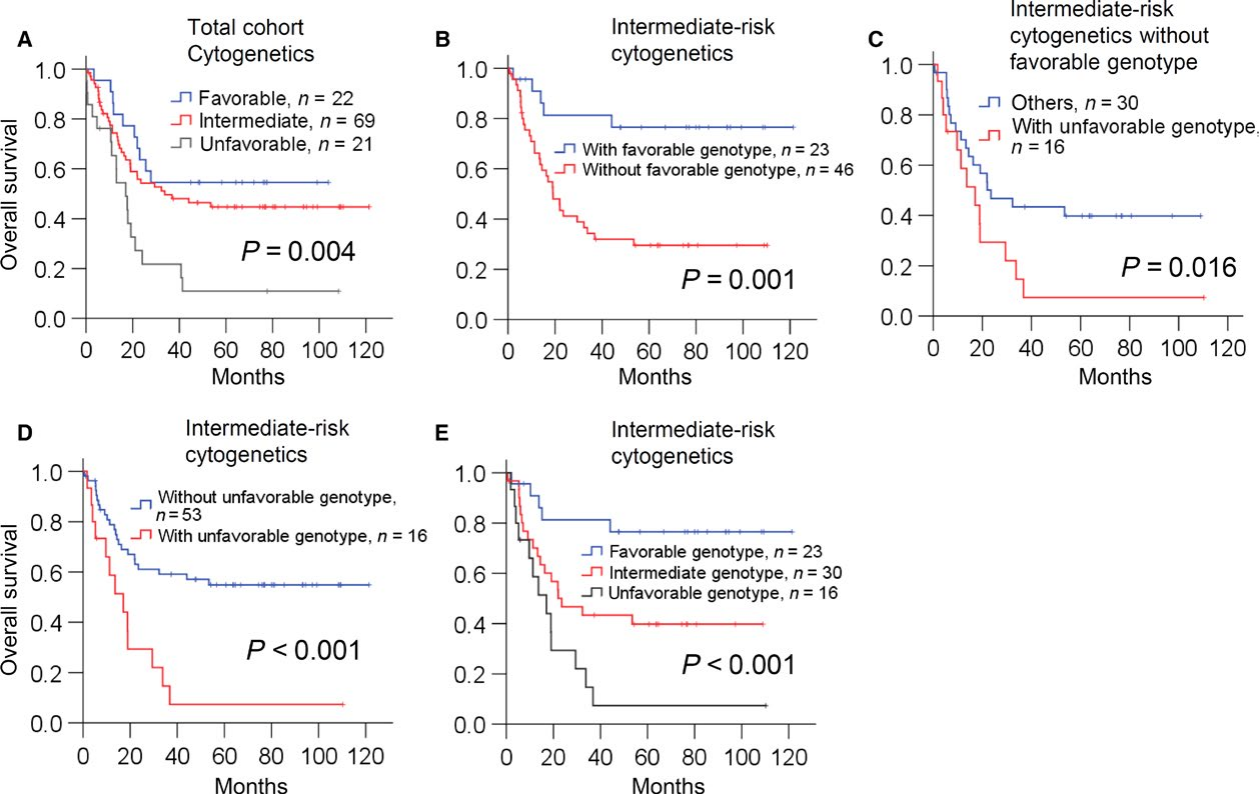
Overall survival estimated by Kaplan–Meier analysis according to (A) cytogenetic risks in the entire cohort. (B) favorable genotype in patients with intermediate‐risk cytogenetics. (C) unfavorable genotype in patients with intermediate‐risk cytogenetics. (D) unfavorable genotype in patients with intermediate‐risk cytogenetics without favorable genotype. (E) revised classification system in patients with intermediate‐risk cytogenetics.

The median follow‐up for the entire cohort was 23.0 months, and 49 surviving patients were followed up for 75.8 months. The 5‐year OS rate was 40.7% [95% CI: 31.6–50.1%]. In the conventional karyotype stratification, the 5‐year OS for patients with favorable‐, intermediate‐, and unfavorable‐risk cytogenetics was 54.5%, 44.7%, and 10.9%, respectively (Fig. 3A, *P* = 0.004).

In order to know whether NGS could be a suitable method to identify mutations for serving clinical prognostic indicators in AML patients, we first examined the relationship between survival and mutation profile of the 40 genes, most of which had been reported to be associated with AML prognosis (Fig. 1, Table 1 and Table S4)^3^. Univariate analysis for all 112 patients revealed that *U2AF1* mutation was associated with a significantly worse OS (HR = 4.293, 95% CI: 1.322–13.94, *P *=* *0.015). Mutation of other genes, including *KIT*,* PHF6*,* TP53*,* RUNX1*,* TET2*,* ASXL1*, and *FLT3*‐ITD, were associated with a nonsignificant trend of reduced OS, whereas mutation of several genes, including *CEBPA* ^double mutation^, *IDH1*,* IDH2*, and *NPM1* were trended to correlate with a prolonged survival (Table 1). In the multivariate analysis (Table 1), the independent poor risk factors were karyotypes, mutations in *TET2*,* PHF6*, and *KIT*. The factor of *NPM1* mutation in the absence of *FLT3*‐ITD (*NPM1* ^mutation^/*FLT3*‐ITD^negative^) was independently correlated with better prognosis. Mutation of *IDH2* or *CEBPA* ^double mutation^ was associated with prolonged OS (Table 1).

**Table 1:** Cox regression hazard analysis on the overall survival in the entire cohort

Variables	*N*	Univariate		Multivariate	
		HR (95% CI)	*P*‐value	HR (95% CI)	*P*‐value
Karyotypes					
Favorable	22	Reference		Reference	
Intermediate	69	1.380 (0.685–2.782)	0.368	4.339 (1.559–12.076)	0.005
Unfavorable	21	3.147 (1.433–6.909)	0.004	7.024 (2.340–21.088)	0.001
Genetic alterations					
CEBPA*	7	0.410 (0.100–1.679)	0.215	0.378 (0.089–1.610)	0.188
* DNMT3A*	14	0.460 (0.184–1.148)	0.096	0.622 (0.188–2.051)	0.435
* IDH2*	14	0.481 (0.192–1.200)	0.117	0.307 (0.083–1.135)	0.077
* IDH1*	4	0.771 (0.188–3.154)	0.718	3.651 (0.747–18.858)	0.110
* GATA2*	7	0.839 (0.263–2.676)	0.767	0.291 (0.055–1.530)	0.145
* NPM1*	17	0.945 (0.450–1.985)	0.882	–	–
* WT1*	13	0.987 (0.425–2.291)	0.976	1.074 (0.438–2.635)	0.876
* FLT3*‐ITD	24	1.223 (0.684–2.186)	0.497	–	–
* ASXL1*	18	1.242 (0.648–2.381)	0.514	1.089 (0.524–2.264)	0.819
* TET2*	12	1.648 (0.784–3.462)	0.188	3.740 (1.598–8.750)	0.002
* RUNX1*	7	1.815 (0.781–4.220)	0.166	2.037 (0.821–5.050)	0.125
* TP53*	9	2.043 (0.816–5.113)	0.127	1.916 (0.628–5.842)	0.253
* PHF6*	3	2.061 (0.645–6.584)	0.223	6.016 (1.255–28.844)	0.025
* KIT*	5	2.429 (0.965–6.117)	0.060	12.131 (3.175–46.358)	<0.001
* U2AF1*	3	4.293 (1.322–13.94)	0.015	6.575 (0.987–43.815)	0.052
* NPM* ^*+*^ */FLT* ^*‐*^	10	0.662 (0.240–1.824)	0.425	0.225 (0.059–0.855)	0.028

HR, hazard ratio; ITD, internal tandem duplication.

CEBPA*: *CEBPA* ^double mutation^; *NPM* ^*+*^ */FLT* ^*‐*^: *NPM1* ^mutation^/*FLT3*‐ITD^negative^

### Analysis of the relationship between other genes and survival

Beyond the 40 genes analyzed, univariate analysis for the other 220 genes showed that *C5*,* GRIK2*,* MYO5B NMUR2*,* TOP3B*,* DOCK2*,* MAP2 KRT79*, and *HYDIN* might be associated with survival (Table S5). Patient number of other genes was too limited to analyze the survival value (less than 5% of total cohort); only the number of cases with *MYO5B*,* KRT79*, and *HYDIN* were enough. *MYO5B* (HR = 2.661, 95% CI: 1.064–6.651) was associated with significantly reduced OS, while *KRT79* and *HYDIN* were trended to correlate with a better survival. To avoid incidental statistical significance in our cohort, we then used TCGA dataset to evaluate their potential prognostic impact 15. However, there were only 2, 2, and 1 patients with *HYDIN*,* KRT79* and *MYO5B* in TCGA dataset, respectively. In addition, the most important value of genetic mutation was to determine the prognosis in patients with intermediate‐risk cytogenetics; these three genes did not affect those patients’ survival. This result indicated that determination of the prognosis was still based on traditional cytogenetics and current known genes.

### Prognostic value of genetic mutations in AML with intermediate‐risk cytogenetics

Among the 69 patients with intermediate‐risk cytogenetics, multivariate analysis showed that patients with *IDH2* mutation, *CEBPA* ^double mutation^, or *NPM1* ^mutation^/*FLT3*‐ITD^negative^ trended to have prolonged OS (all HR<0.5 and *P* < 0.15). Thus, 23 patients with intermediate‐risk cytogenetics who had at least one of the above genetic alterations had a significantly better survival (Fig. 3B, *P* = 0.001) as compared with the 46 patients who did not have these mutations.

Subgroup analysis of the 46 patients without favorable genotypes revealed that patients (*n* = 16) with *TET2*,* RUNX1*,* ASXL1*, or *DNMT3A* had a trend of reduced OS (Table S7, all HR>1.5). Based on the poor trend of OS and previous studies reporting them as poor prognostic factors 3, we grouped these genetic mutations as an unfavorable subclass. These 16 patients had a significantly inferior OS (Fig. 3C, *P* = 0.016) among the 46 patients without favorable genotypes, and among the overall 69 patients with intermediate‐risk cytogenetics (Fig. 3D, *P* < 0.001).

For the 69 patients with intermediate‐risk cytogenetics, we classified them into three groups according to genotype: mutation of *IDH2*,* CEBPA* ^double mutation^, or *NPM1* ^mutation^ in the absence of *FLT3*‐ITD as a favorable genotype, mutation of *TET2*,* RUNX1*,* ASXL1*, or *DNMT3A* as an unfavorable genotype, and the remaining was the intermediate genotype (Table 2). The above results indicated that AML patients with intermediate‐risk cytogenetics could be classified into three risk groups according to genotype (Fig. 3E, *P* < 0.001).

**Table 2:** Cox regression hazard analysis of combined gene group on the overall survival in the patients with intermediate‐risk cytogenetics

Group	*N*	Univariate	
		HR (95% CI)	*P*‐value
Mutants *NPM1*,* IDH2*, or *CEBPA* ^double mutation^ in the absence of *FLT3*‐ITD	23	Reference	
Others	30	3.293 (1.212–8.943)	0.019
Mutants *TET2*,* RUNX1*,* AXSL1*, or *DNMT3A*	16	7.735 (2.740–21.833)	<0.001

HR, hazard ratio; ITD, internal tandem duplication.

### Poor prognostic impact of *KIT* mutation in favorable‐risk cytogenetic AML

Prior studies reported *KIT* mutation as a poor factor in favorable‐risk cytogenetic AML 32. In our cohort, five patients had a *KIT* mutation, and all of them had favorable‐risk cytogenetic AML. Among the 22 patients with favorable‐risk cytogenetic AML, patients with *KIT* mutation had significantly poorer OS (HR = 7.002, 95% CI: 1.925–25.467, *P *=* *0.003).

### Integrated classification of cytogenetic and genetic profiling

Among the 69 patients with intermediate**‐**risk cytogenetics, 23 patients had the favorable genotype and their 5‐year survival was 76.5% (95% CI: 58.5–94.5%), similar to the prognosis of patients with favorable‐risk cytogenetics (5‐year OS, favorable cytogenetics vs. favorable genotype of intermediate cytogenetics, *P *=* *0.161). For another 16 patients with the unfavorable genotype, the 5‐year OS was also similar to the OS of patients with unfavorable cytogenetics (5‐year OS, unfavorable cytogenetics vs. unfavorable genotype of intermediate cytogenetics, *P *=* *0.674). In addition, five patients with favorable‐risk cytogenetic AML plus *KIT* mutation had a reduced OS compared with those without *KIT* mutation, and might be considered as intermediate risk.

**Figure 4:**
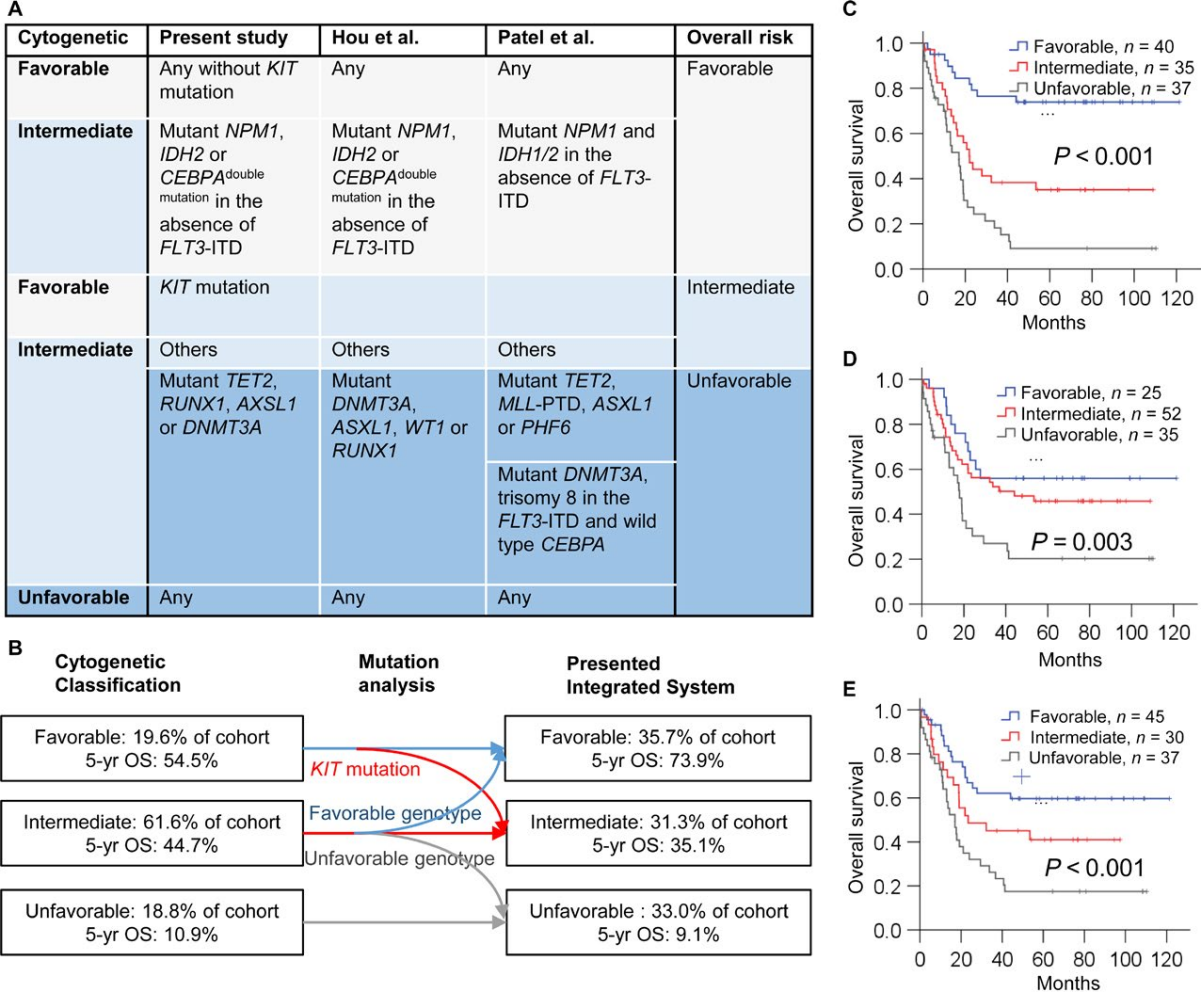
Revised risk classification of acute myeloid leukemia (AML) patients. (A) Mutation profiling of this study, Hou et al.^7^, and Patel et al.^3^ for risk stratification of AML patients. (B) Effect of the mutation profiling on the conventional cytogenetic risk. Patients with intermediate‐risk cytogenetic AML who have markedly divergent prognoses are reassigned into the appropriate risk groups according to their mutation profiles. Patients with favorable‐risk cytogenetics and *KIT* mutation are reclassified as intermediate risk. Overall survival estimated by Kaplan–Meier analysis according to (C) the current integrated stratification system, (D) revised risk system of Patel et al., and (E) risk stratification system of Hou et al.

The above results allowed us to develop a prognostic classification according to integration of the genetic mutation analysis and cytogenetic data (Fig. 4A). The 5‐year OS rate of patients with the new favorable‐risk, intermediate‐risk, and unfavorable‐risk groups was 73.9%, 35.1%, and 9.1%, respectively (Fig. 4B and C, *P *< 0.001). The univariate HR of the intermediate‐risk patients was 3.49 (95% CI: 1.64–7.34; intermediate‐risk cytogenetics: 1.38, 95% CI: 0.69–2.78) and HR of the unfavorable‐risk group was 6.77 (95% CI: 3.28–13.98; unfavorable cytogenetics: 3.15, 95% CI: 1.43–6.90), indicating that risk stratification using the integrated system was more clinically informative than that using cytogenetics alone (Figs. 3A vs. and 4C). In addition, integrated risk classification significantly reduces the proportion of intermediate‐risk patients from about 60% to 25%.

## Discussion

Our results demonstrate that integration of cytogenetic and genetic mutation profiles, with parallel sequencing by NGS, can improve the risk stratification for AML patients, especially for patients with intermediate‐risk cytogenetics.

The ultimate goal of cancer therapy is to establish precision medicine for guiding the best treatment and maximizing patient survival 33. For patients with unfavorable‐risk cytogenetics, treating with allogeneic HSCT in their first CR is currently evident strategy to improve their survival; however, the survival benefit from this strategy is not shown in patients with favorable‐risk cytogenetics 6. For unfavorable genotypes, such as *NPM1* ^negative^/*FLT3*‐ITD^positive^ or *RUNX1* mutation, allogeneic HSCT can prolong patient survival 16, 34, 35. These facts indicate that early identification of poor‐risk patients can guide treatment. Risk classification of AML based on the traditional cytogenetic study is not good enough because most patients belong to intermediate‐risk cytogenetics. Recent studies show that using a list of genetic mutations can establish a prognostic classification to classify AML patients with intermediate‐risk cytogenetics into more definitive prognostic groups 36. European LeukemiaNet first stratified AML patients with normal cytogenetics into two risk groups using the mutation status of *NPM1*,* FLT3*, and *CEBPA* 8. Studies of Patel et al. and Hou et al. utilized 10 and 8 genes to classify intermediated cytogenetics into favorable, intermediate, and unfavorable subgroups 3, 7. In this study, we comprehensively sequenced 260 genes; nine genes were integrated with cytogenetics to develop a revised risk classification (Fig. 4A). For 69 patients with intermediate‐risk cytogenetics, the risk for 23 and 16 patients was revised as favorable and unfavorable, respectively. We also evaluated the two other risk stratification systems on the basis of mutation profiling in our cohort; the survival difference between three risk groups was more significant (Fig. 4D and E) compared with cytogenetic stratification alone (Fig. 3A). These facts suggest that utilizing mutation profiles of multiple genes could classify patients with intermediate‐risk cytogenetics into more accurate risk classification groups so as to significantly reduce the number of AML patients classified as intermediate risk.

In a comparison of our integrated classification and prior two studies, the mutational profiles of each risk classification were similar but not totally the same (Fig. 4A). Patel et al. reported that the *IDH1*/*2* mutation is a favorable prognostic factor for AML with mutated *NPM1* without *FLT3*‐ITD 3; our cohort and Hou et al. did not find *IDH1* mutation as a favorable risk factor. *IDH1* mutation was reported to be associated with unfavorable risk or did not affect disease outcome in other AML studies 37, 38. These results indicate that the prognostic value of *IDH1* mutation is controversial. For *IDH2*, all three studies revealed that *IDH2* mutation is associated with favorable risk only in the absence of *FLT3*‐ITD (Fig. 4A). However, a survival analysis reported that the *IDH2* R140 mutation is associated with favorable prognosis and R172 with poor prognosis 39. Other investigations revealed that AML patients with *IDH* mutation respond better to treatment with hypomethylating agents 40, 41. These data indicate that risk stratification using *IDH* mutation may need to consider the therapeutic agents, intrinsic mutation site, and extrinsic genetic modifiers. Another difference in the favorable‐risk genotype is *CEBPA* ^double mutation^, which was found in our study, Hou et al. and in other studies 7, 42, 43, but the prognostic relevance was not reported by Patel et al. 3. Comparison of the unfavorable genotypes indicated that mutants *ASXL1*,* TET2*,* and DNMT3A* were identified as unfavorable risk factors in the three studies. Mutation of *RUNX1* was considered as a poor factor in previous reports and in this study 7, 44. Mutant *PHF6* was not seen in our patients with intermediate‐risk cytogenetics, and all three patients carrying this mutation were in the favorable‐ or unfavorable‐risk cytogenetic group and died from the disease. Although the mutation profiles of favorable and unfavorable genotypes across the three studies are not the same, a substantial proportion of the molecular profiles are similar. The differences might be caused by different enrollment criteria and treatment agents, such as the high‐dose daunorubicin used in patients of Patel's study versus the standard‐dose anthracycline used in our patients. In the three studies, a total of 12 genes were analyzed for risk stratification (Fig. 4A). Parallel sequencing using NGS is a good strategy to handle the testing of multiple genes and can provide a rapid and accurate risk classification system for the clinical management of AML patients 14.

This study contained several limitations. This study was retrospective and chemotherapy regimens were not stringently the same, but all patients received Idarubicin and Cytarabine (*7* + *3*) as induction chemotherapy, followed by high‐dose Cytarabine‐based consolidation. Therefore, we considered this cohort to be appropriate for analyzing genetic values in the prognosis. We also tried to search for new genetic factors associated with patients’ survival, especially for patients with intermediate‐risk cytogenetics; only nine new genes might be related to prognosis (Table S5). However, case numbers of other gene were too limited to analyze their real effect and the prognostic impact cannot be validated in TCGA dataset, indicating no new genetic mutations significantly affecting AML prognosis. Several recent studies found *TP53* mutation was associated with a poor survival, especially predicting the worst outcome in patients with unfavorable cytogenetics 45, 46, 47. In our cohort, *TP53* mutation was associated with an inferior trend of OS among the whole cohort (Table 1) and usually co‐occurred with unfavorable cytogenetics. *TP53* mutation predicted significantly worse OS in patients with unfavorable cytogenetics (*P *=* *0.006), but did not play a role in other cytogenetics. In addition to genetic factors, clinical factors, such as age, were reported to be associated with patients’ survival 48. In this study, age did not significantly affect patients’ outcome and the genetic value in the multivariate analysis (Table S6). These indicated that currently known genes were the most important factors predicting survival.

In conclusion, with early assessment of cytogenetics and mutational profiling, AML patients can be managed by their real risk to reduce the mortality that results from unfavorable cytogenetics or genotypes. Therefore, accurate and rapid molecular diagnosis is important in AML patients. To achieve this goal, our study demonstrates that NGS‐based multi‐gene sequencing is clinically applicable and can be an effective means of establishing an accurate risk stratification system for guiding therapeutic decisions.

## Conflicts of Interests

The authors declare that they have no conflicts of interests.

## Supporting information


**Table S1.** Genes analyzed in this study.**Table S2.** Clinical and laboratory characteristics of AML patients.
**Table S3.** Pearson chi‐square analysis of pairwise gene–gene associations among the 40 genes involved in leukemogenesis.
**Table S4.** Univariate analysis of 40 genes with respect to overall survival in the entire cohort.
**Table S5.** List of genes with positive correlation (*P* ≤ 0.01) to OS in the entire cohort and their impact on patients with intermediated cytogenetics.**Table S6.** Cox regression hazard analysis of each gene with age factor on the overall survival in patients with intermediate‐risk cytogenetics.
**Table S7.** Multivariate analysis of overall survival of patients with intermediate‐risk cytogenetics without favorable genotype (*N* = 37).
**Figure S1.** Detection of *FLT3* ITDs by GATK (HaplotypeCaller) and Pindel.
