# Supplementary material for: A targeted next‐generation sequencing in the molecular risk stratification of adult acute myeloid leukemia: implications for clinical practice
Source: Cancer Med. 2017 Jan 10;6(2):349–60. doi: 10.1002/cam4.969 (PMC5313641; doi:10.1002/cam4.969)
Supplement: Supplementary file 1 — Table S1. Genes analyzed in this study.Table S2. Clinical and laboratory characteristics of AML patients. Table S3. Pearson chi‐square analysis of pairwise gene–gene associations among the 40 genes involved in leukemogenesis. Table S4. Univariate analysis of 40 genes with respect to overall survival in the entire cohort. Table S5. List of genes with positive correlation (P ≤ 0.01) to OS in the entire cohort and their impact on patients with intermediated cytogenetics.Table S6. Cox regression hazard analysis of each gene with age factor on the overall survival in patients with intermediate‐risk cytogenetics. Table S7. Multivariate analysis of overall survival of patients with intermediate‐risk cytogenetics without favorable genotype (N = 37). Figure S1. Detection of FLT3 ITDs by GATK (HaplotypeCaller) and Pindel. [file CAM4-6-349-s001.docx]

**Supplementary Table 1.** Genes analyzed in this study

| ABCG2 | DIS3 | HIVEP1 | MYH4 | SCML2 |
| --- | --- | --- | --- | --- |
| ABTB1 | DLC1 | HMCN1 | MYO5B | SCN1A |
| ADAM11 | DNAH5 | HNRNPK | MYOC | SEMA3A |
| ADCY5 | DNAH9 | HSP90B3P | MYOM3 | SEMA4A |
| AKAP13 | DNAI1 | HYDIN | NALCN | SETBP1 |
| ALPK3 | DNMT3A | IDH1 | NAV1 | SF3B1 |
| ANK2 | DNMT3B | IDH2 | NF1 | SHC1 |
| APOB | DOCK2 | IGHG3 | NLRC4 | SHROOM2 |
| ARAP2 | DRD2 | IKZF4 | NMUR2 | SI |
| ASXL1 | DSCAM | ILDR1 | NPM1 | SLC12A3 |
| ATG16L1 | DST | ITPR3 | NR2E1 | SLC39A5 |
| ATP10B | DYNC2H1 | JAK1 | NRAS | SMC1A |
| ATP2B3 | DYSF | JAK2 | NRXN3 | SMC3 |
| BCOR | E2F8 | JAK3 | NTRK3 | SMG1 |
| BMPER | EDIL3 | KCNA4 | OR11H12 | SPEG |
| BOD1L | EED | KCNH2 | OR13H1 | SPEN |
| BSN | EEF1A1P29 | KCNK13 | OR8B12 | SRSF2 |
| C10orf118 | EGFR | KCNQ2 | P2RY2 | ST13P13 |
| C10orf28 | EPHA10 | KCNT1 | PCDHA13 | STAG2 |
| C17orf97 | EPPK1 | KCNU1 | PCDHA6 | STC2 |
| C5 | ETV6 | KDM3B | PCDHB1 | STRN |
| CACNA1B | EZH2 | KDM6A | PCDHB18 | SUZ12 |
| CACNA1E | FAM154B | KDR | PDCD2L | SYNGAP1 |
| CACNA1G | FAM40B | KIAA0240 | PHACTR1 | SYT15 |
| CACNA2D3 | FAM47A | KIAA1267 | PHF6 | TCEAL3 |
| CADM2 | FAM57B | KIAA1529 | PHIP | TCEAL6 |
| CADPS | FAM5C | KIAA1683 | PKD1L2 | TET1 |
| CALR | FAM65A | KIF2B | PKD2L1 | TET2 |
| CBFB | FAM70B | KIT | PKHD1 | THRAP3 |
| CBL | FCGBP | KRAS | PKHD1L1 | TMEM104 |
| CCDC67 | FKBP8 | KRT19 | PLCE1 | TNC |
| CD74 | FLG | KRT79 | PLEKHH1 | TOP3B |
| CEBPA | FLRT2 | KSR2 | PPP1R3A | TP53 |
| CECR2 | FLT1 | LNX1 | PRAMEF2 | TRA2B |
| CELSR3 | FLT3 | LRBA | PRPF4B | TRPM3 |
| CHD4 | FOXP1 | LRIT1 | PRPF8 | TTBK1 |
| CLEC18B | FREM2 | LRP1B | PSME4 | TUBA3C |
| CMYA5 | FRYL | LRRC4 | PTCH1 | TYK2 |
| CNTN5 | GALNTL4 | MAGI2 | PTPN11 | U2AF1 |
| CNTNAP4 | GAS6 | MAP1B | PTPRN | UNC5B |
| COL12A1 | GATA2 | MAP2 | PTPRT | USP9X |
| COL5A3 | GBP4 | MED12 | RAD21 | VARS2 |
| CRISPLD1 | GIGYF2 | MEFV | RBBP4 | VCAN |
| CROCC | GJB3 | MEGF8 | RFC3 | WAC |
| CSMD1 | GPR112 | MIR142 | RIMS1 | WT1 |
| CSMD3 | GPR183 | MLL3 | RNF213 | XIRP1 |
| CUEDC1 | GRID1 | MROH5 | RUNX1 | ZBTB33 |
| CUL1 | GRIK2 | MTA2 | RUNX1T1 | ZC3H18 |
| DCHS2 | GRIK4 | MTMR8 | RYR1 | ZNF687 |
| DCLK1 | GRM3 | MTUS2 | RYR3 |  |
| DDR2 | GRM8 | MUC16 | SBF1P1 |  |
| DDX11 | GSTK1 | MUC5B | SCAF8 |  |
| DDX41 | HECW1 | MYC | SCARB1 |  |

Adapted from https://sg.idtdna.com/pages/products /nextgen/target-capture/xgen-lockdown-panels/xgen-aml-cancer-panel

**Supplementary Table 2.** Clinical and laboratory characteristics of AML patients

| Variables | Total (n=112) |
| --- | --- |
| Age, mean ± SD | 42.6 ± 14.8 |
| Sex |  |
| Male | 67 |
| Female | 45 |
| Lab data |  |
| WBC, 10^6^/L, mean ± SD | 53.4 ± 81.6 |
| Hb, g/L, mean ± SD | 7.6 ± 2.8 |
| Platelet, 10^6^/L, mean ± SD | 67.7 ± 132.7 |
| Blast, 10^6^/L, mean ± SD | 35.5 ± 74.8 |
| LDH, IU/L, mean ± SD | 548.0 ± 694.2 |
| FAB type |  |
| M0 | 5 |
| M1 | 21 |
| M2 | 37 |
| M3 | 9 |
| M4 | 21 |
| M5 | 6 |
| M6 | 2 |
| M7 | 1 |
| Undetermined | 10 |
| Karyotype |  |
| Favorable | 22 |
| Intermediate | 69 |
| Unfavorable | 21 |
| Allogeneic HSCT |  |
| Received | 19 |
| Not received | 93 |

*Favorable, t(8;21), inv(16); unfavorable, -7, del(7q), -5, del(5q), 3q abnormality, complex abnormalities; Intermediate, normal karyotype and other abnormalities.

**Supplementary Table 3.** Person chi square analysis of pairwise gene-gene associations among the 40 genes involved in leukemogenesis

**Supplementary Table 4.** Univariate analysis of 40 genes with respect to overall survival in the entire cohort

| Gene | HR | upper limit | lower limit | P value |  | Gene | HR | upper limit | lower limit | P value |
| --- | --- | --- | --- | --- | --- | --- | --- | --- | --- | --- |
| *ASXL1* | 1.242 | 0.648 | 2.381 | 0.514 |  | *MTA2* | 0.049 | 0.000 | 555.837 | 0.526 |
| *CBFB* | 1.581 | 0.384 | 6.504 | 0.526 |  | *MYC* | - | - | - | - |
| *CEBPA** | 0.410 | 0.100 | 1.679 | 0.215 |  | *NPM1* | 0.945 | 0.450 | 1.985 | 0.882 |
| *DNMT3A* | 0.460 | 0.184 | 1.148 | 0.096 |  | *NRAS* | 0.048 | 0.000 | 217.332 | 0.480 |
| *DNMT3B* | 0.873 | 0.213 | 3.573 | 0.850 |  | *PHF6* | 2.061 | 0.645 | 6.584 | 0.223 |
| *EED* | - | - | - | - |  | *PRPF8* | 0.667 | 0.163 | 2.729 | 0.573 |
| *ETV6* | 1.259 | 0.308 | 5.154 | 0.749 |  | *PTPN11* | 0.397 | 0.097 | 1.627 | 0.199 |
| *EZH2* | 1.065 | 0.334 | 3.398 | 0.915 |  | *RBBP4* | 0.547 | 0.076 | 3.951 | 0.550 |
| *FLT3_ITD* | 1.227 | 0.687 | 2.193 | 0.489 |  | *RUNX1* | 1.815 | 0.781 | 4.220 | 0.166 |
| *GATA2* | 0.839 | 0.263 | 2.676 | 0.767 |  | *SCML2* | - | - | - | - |
| *IDH1* | 0.771 | 0.188 | 3.154 | 0.718 |  | *SF3B1* | 0.481 | 0.067 | 3.470 | 0.468 |
| *IDH2* | 0.481 | 0.192 | 1.200 | 0.117 |  | *SRSF2* | 1.290 | 0.178 | 9.328 | 0.801 |
| *JAK1* | 1.698 | 0.415 | 6.951 | 0.462 |  | *SUZ12* | 1.102 | 0.269 | 4.516 | 0.892 |
| *JAK2* | 0.869 | 0.273 | 2.773 | 0.813 |  | *TET1* | 1.559 | 0.379 | 6.420 | 0.538 |
| *JAK3* | 1.391 | 0.557 | 3.471 | 0.480 |  | *TET2* | 1.648 | 0.784 | 3.462 | 0.188 |
| *KDM3B* | 1.120 | 0.274 | 4.582 | 0.875 |  | *TP53* | 2.043 | 0.816 | 5.113 | 0.127 |
| *KDM6A* | 1.025 | 0.321 | 3.271 | 0.967 |  | *TRA2B* | - | - | - | - |
| *KIT* | 2.429 | 0.965 | 6.117 | 0.060 |  | *U2AF1* | 4.293 | 1.322 | 13.940 | 0.015 |
| *KRAS* | 1.470 | 0.202 | 10.680 | 0.703 |  | *WT1* | 0.987 | 0.425 | 2.291 | 0.976 |
| *MLL3* | - | - | - | - |  | *ZBTB33* | 2.346 | 0.322 | 17.108 | 0.400 |

**Supplementary Table 5.** List of gene with positive correlation (p≤0.01) to OS in the entire cohort and their impact in patients with intermediated-cytogenetics

|  | Total cohort | | | | | Intermediate cytogenetics | | | | |
| --- | --- | --- | --- | --- | --- | --- | --- | --- | --- | --- |
| Gene | N | HR | upper limit | lower limit | P value | N | HR | upper limit | lower limit | P value |
| *C5* | 1 | 110.50 | 6.91 | 1766.61 | 0.001 | 0 | - |  |  |  |
| *GRIK2* | 2 | 6.60 | 1.56 | 27.84 | 0.010 | 1 | - |  |  |  |
| *MYO5B* | 7 | 2.66 | 1.06 | 6.65 | 0.036 | 4 | 1.13 | 0.27 | 4.70 | 0.871 |
| *NMUR2* | 5 | 2.39 | 0.96 | 5.99 | 0.062 | 3 | 1.29 | 0.31 | 5.39 | 0.724 |
| *TOP3B* | 4 | 2.56 | 0.93 | 7.05 | 0.07 | 0 | - |  |  |  |
| *DOCK2* | 5 | 2.68 | 0.84 | 8.59 | 0.096 | 3 | 0.987 | 0.14 | 7.23 | 0.990 |
| *MAP2* | 5 | 2.68 | 0.84 | 8.59 | 0.096 | 3 | 0.987 | 0.14 | 7.23 | 0.990 |
| *KRT79* | 8 | 0.30 | 0.07 | 1.24 | 0.097 | 4 | 0.63 | 0.09 | 4.57 | 0.644 |
| *HYDIN* | 6 | 0.221 | 0.031 | 1.591 | 0.100 | 4 | 0.044 |  | 0 | 0.280 |

**Supplementary Table 6.** Cox regression hazard analysis of each gene with age factor on the overall survival in the patients with intermediate-risk cytogenetics

|  | HR | 95.0% CI | | P value |
| --- | --- | --- | --- | --- |
|  |  | Lower | Upper |  |
| TP53 | 0.655 | 0.063 | 6.823 | 0.723 |
| WT1 | 0.731 | 0.153 | 3.501 | 0.695 |
| DNMT3A | 0.969 | 0.322 | 2.914 | 0.955 |
| TET2 | 2.056 | 0.729 | 5.797 | 0.173 |
| IDH1 | 2.160 | 0.208 | 22.412 | 0.519 |
| IDH2 | 0.242 | 0.048 | 1.215 | 0.085 |
| *NPM^+^/FLT^-^* | 0.325 | 0.072 | 1.458 | 0.142 |
| *RUNX1* | 2.151 | 0.780 | 5.931 | 0.139 |
| *CEBPA** | 0.198 | 0.027 | 1.479 | 0.114 |
| *ASXL1* | 1.157 | 0.244 | 5.491 | 0.854 |
| age | 1.017 | 0.991 | 1.043 | 0.199 |

*CEBPA**: *CEBPA*^double mutation^; *NPM^+^/FLT^-^*: *NPM1*^mutation^/*FLT3*-ITD^negative^

**Supplementary Table 7.** Multivariate analysis of overall survival of patients with intermediate-risk cytogenetics without favorable genotype (N=37)

|  | **Multivariate** |  |
| --- | --- | --- |
| **Variables** | **HR (95% CI)** | **p-value** |
| *TP53* | 0.322 (0.034-3.065) | 0.324 |
| *WT1* | 0.787 (0.181-3.417) | 0.750 |
| *ASXL1* | 2.619 (0.665-10.473) | 0.173 |
| *TET2* | 3.163 (1.016-9.853) | 0.047 |
| *RUNX1* | 1.665 (0.529-5.244) | 0.384 |
| *DNMT3A* | 2.170 (0.705-6.680) | 0.177 |

**Supplementary figure 1**

**
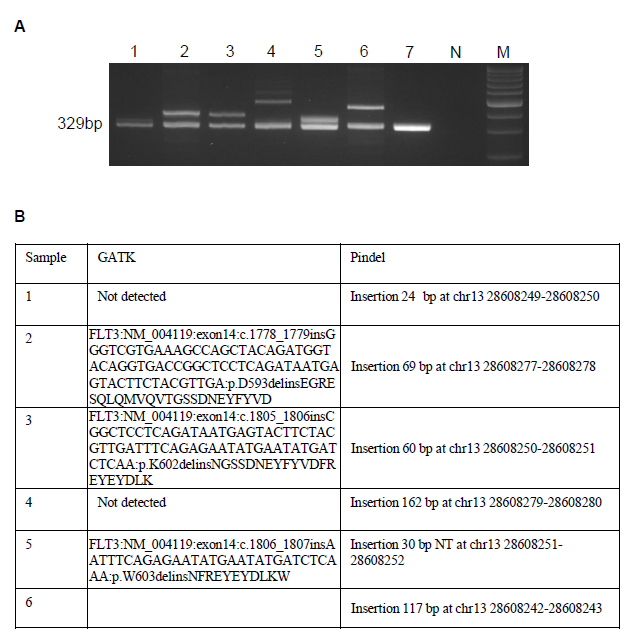
**

Detection of *FLT3* ITDs by GATK (HaplotypeCaller) and Pindel. (A) Gel electrophorectogram of polymerase chain reaction (PCR) products amplified by a pair of primers: Forward: GCAATTTAGGTATGAAAGCCAGC and Reverse: CTTTCAGCATTTTGACGGCAACC. Lane 1-6 represented the PCR product amplified from AML DNA samples with FLT3-ITD and the size of DNA fragments greater than 329 bp stand for the existence of ITD; land 7 denotes the PCR product (329 bp) from a wild-type sample and lane M stands for the 100 bp size marker.(B) FLT3-ITD was detected by GATK (HaplotypeCaller) and Pindel.
